# Functional Outcomes of Primary Hemiarthroplasty for Unstable Intertrochanteric Fractures in the Elderly: A Prospective Observational Study

**DOI:** 10.7759/cureus.54892

**Published:** 2024-02-25

**Authors:** Shravan Peddamadyam, Bodla Arvind Kumar, Reddy Vamsi Krishna Arcot

**Affiliations:** 1 Department of Orthopaedics, Nizam's Institute of Medical Sciences, Hyderabad, IND; 2 Department of Orthopaedics, Nizam's Institute of Medical Sciences, Panjagutta, Hyderabad, IND

**Keywords:** cemented bipolar, elderly, osteoporosis, unstable intertrochanteric fracture, primary hemiarthroplasty

## Abstract

Background and objective

Intertrochanteric fractures pose a growing healthcare challenge among the elderly, demanding effective management strategies. This study addressed the rising incidence of hip fractures, emphasizing the complications associated with traditional nonsurgical approaches. It aimed to explore postoperative functional outcomes and complications associated with primary hemiarthroplasty as an alternative to internal fixation for unstable intertrochanteric fractures in the elderly.

Materials and methods

This study included 20 elderly patients undergoing hemiarthroplasty for unstable intertrochanteric fractures. It evaluated key variables such as patient demographics, comorbidities, fracture characteristics, surgical approach, and postoperative metrics. To perform hemiarthroplasty, we utilized a nonmodular bipolar prosthesis with cement. Postoperative follow-up included an assessment of clinical and radiological parameters, focusing on outcomes and complications.

Results

The mean age of the participants was 71.65 years; it was found that a significant segment of the participants (n=9, 45%) did not have any comorbidities. The surgical outcomes were characterized by minimal blood loss (275 ± 57.35 ml), short hospital stays (6.55± 1.95 days), and satisfactory operative durations (80.25 ± 10.19 minutes). Additionally, 14 (70%) patients did not require blood transfusions. After the surgery, complications were minimal, and there were no cases of deep wound complications, prosthesis dislocations, or deep vein thrombosis. The Harris Hip Scores reflected favorable outcomes in 14 cases (72.7%), with good or excellent hip scores.

Conclusions

Our findings revealed that primary hemiarthroplasty is a reliable and effective strategy for managing unstable intertrochanteric fractures in the elderly, providing stable joints and acceptable complication rates. Early mobilization, facilitated by hemiarthroplasty, mitigates postoperative complications, making it a viable alternative for elderly patients.

## Introduction

The burden of intertrochanteric fractures among the elderly has assumed considerable significance in the healthcare field. Given the demographic changes characterizing the contemporary world, marked by the rise in elderly populations, the incidence of hip fractures, including intertrochanteric fractures, has increased at an unprecedented rate [1,2]. These fractures typically arise from low-energy trauma, such as a fall from a standing height, and are marked by their propensity for instability, causing significant pain, mobility loss, and adverse effects on the overall quality of life in elderly individuals [3].

Before the 1960s, the conventional approach to managing intertrochanteric fractures involved nonsurgical treatments, primarily consisting of traction and extended bed rest. This conservative approach was associated with a prolonged healing process spanning 10-12 weeks, followed by extensive ambulation training [4]. However, this treatment method has been plagued with complications stemming from the extended immobilization of patients, such as the development of decubitus ulcers (pressure sores), urinary tract infections (UTIs), joint contractures, pneumonia, and an elevated risk of thromboembolic events, which collectively contribute to a marked increase in mortality rates [4,5].

Historically, the use of fixed nail plate devices for treating these fractures has resulted in a higher incidence of complications, including cut-outs and fracture displacements. As a result, sliding hip screws have become a more successful and widely used fixation method for these fractures. However, complications have persisted, including head perforations, excessive sliding causing shortening, plate pull-out, and plate breakage, particularly in cases involving unstable fractures. Osteoporosis and instability were found to significantly contribute to suboptimal outcomes in these patients [6]. As a strategic response to these considerations, primary hemiarthroplasty has emerged as an efficacious approach to the management of unstable fractures in elderly patients. This method not only promotes earlier postoperative weight-bearing but also facilitates rapid rehabilitation, all while exhibiting a commendably low failure rate [7]. The objective of the current study is to evaluate the functional outcome of primary hemiarthroplasty for unstable intertrochanteric fractures in the elderly.

## Materials and methods

We employed a prospective study design to conduct the study, which spanned the period from September 2021 to August 2022. The sampling technique included convenient sampling by recruiting 20 patients who underwent hemiarthroplasty for unstable intertrochanteric fractures. The inclusion criteria were individuals aged ≥60 years who presented with unstable intertrochanteric fractures and concurrent osteoporosis. All participants provided written informed consent before their inclusion in the study. Conversely, the exclusion criteria included patients below 60 years of age, those with stable intertrochanteric fractures, and individuals deemed unfit for surgical intervention. Following comprehensive evaluations and preanesthetic assessments, surgeries were performed on an elective basis with a strategic approach to mitigate avoidable anesthetic risks. The collected data included key variables such as age, sex, mode of injury, fracture type, surgical approach, prosthesis utilized, hospital stay duration, time to achieve full weight-bearing, and any recorded complications.

**Figure 1:**
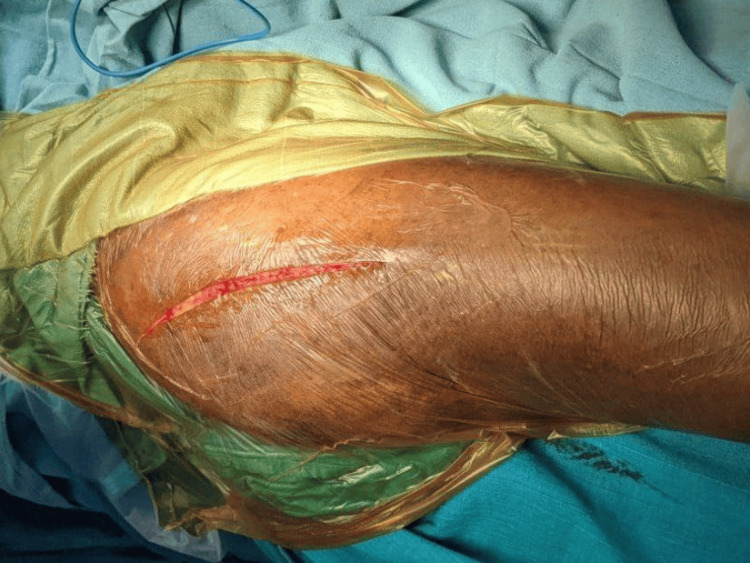
Posterior approach to the hip joint

In this study, a surgical approach involving either a posterior (Moore) or lateral (Hardinge) approach was employed on patients, who were placed in the lateral decubitus position, with the affected limb positioned uppermost (Figure 1).

**Figure 2:**
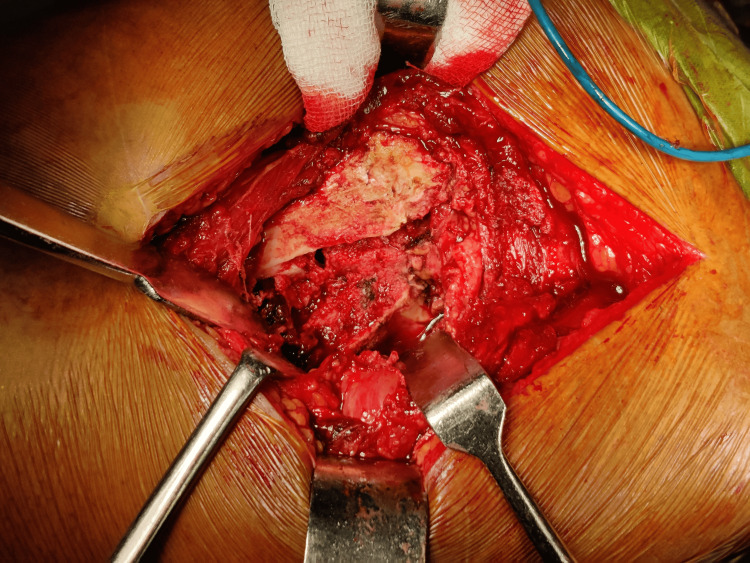
Exposure of fracture fragments

**Figure 3:**
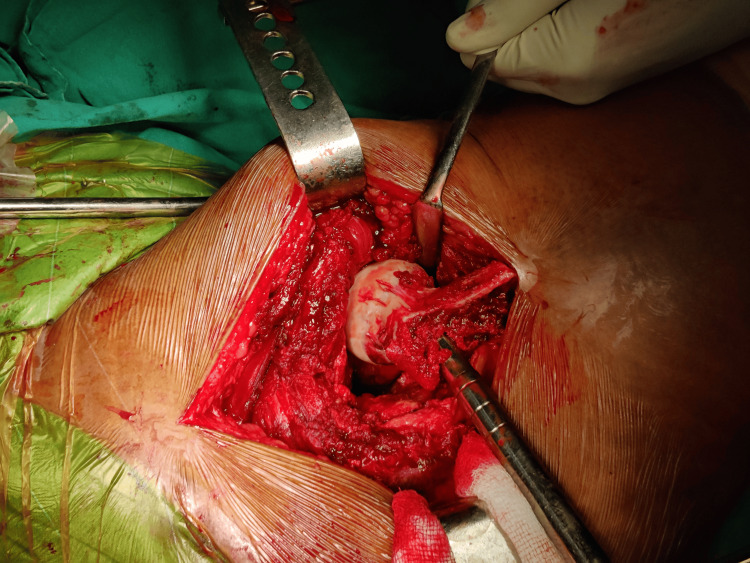
Extraction of femoral head out of acetabulum

**Figure 4:**
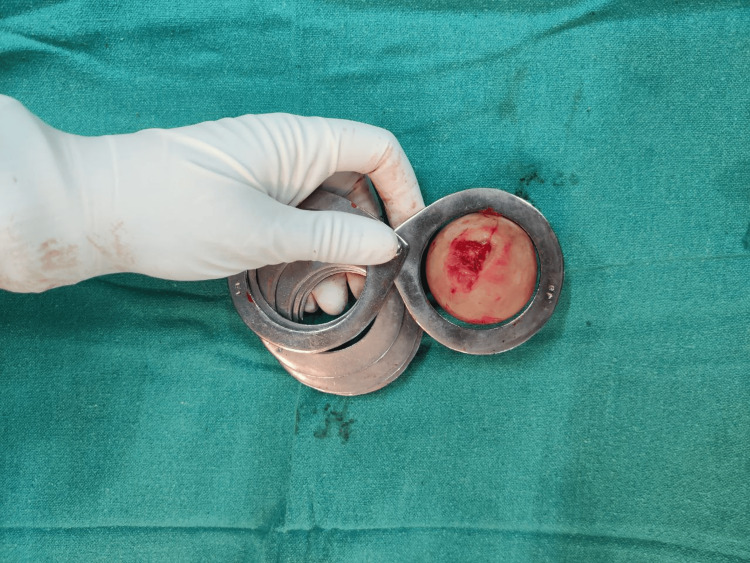
Measurement of the femoral head using a template

Following exposure, fracture identification, and careful levering of the femoral head and neck out of the acetabulum, measurements of the femoral head were performed using a template (Figures 2-4).

**Figure 5:**
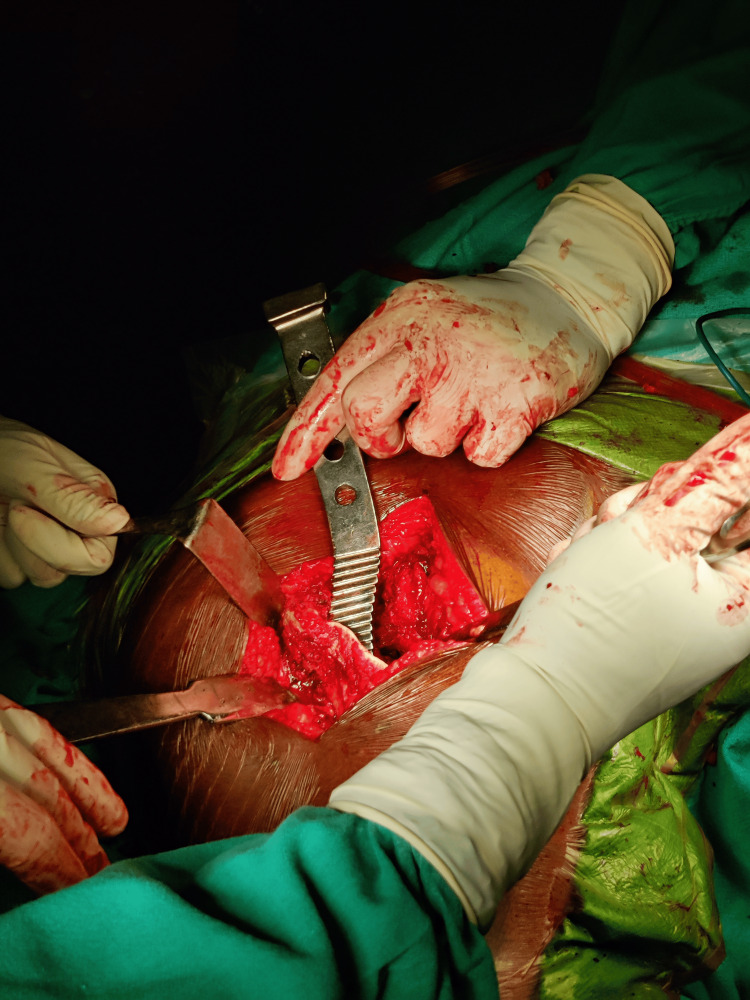
Preparation of the femoral canal using rasp

Complete exposure and clearance of the acetabulum from all soft tissues were ensured. The femoral canal was meticulously prepared, and trial reduction was performed to ascertain the vertical offset (Figure 5).

**Figure 6:**
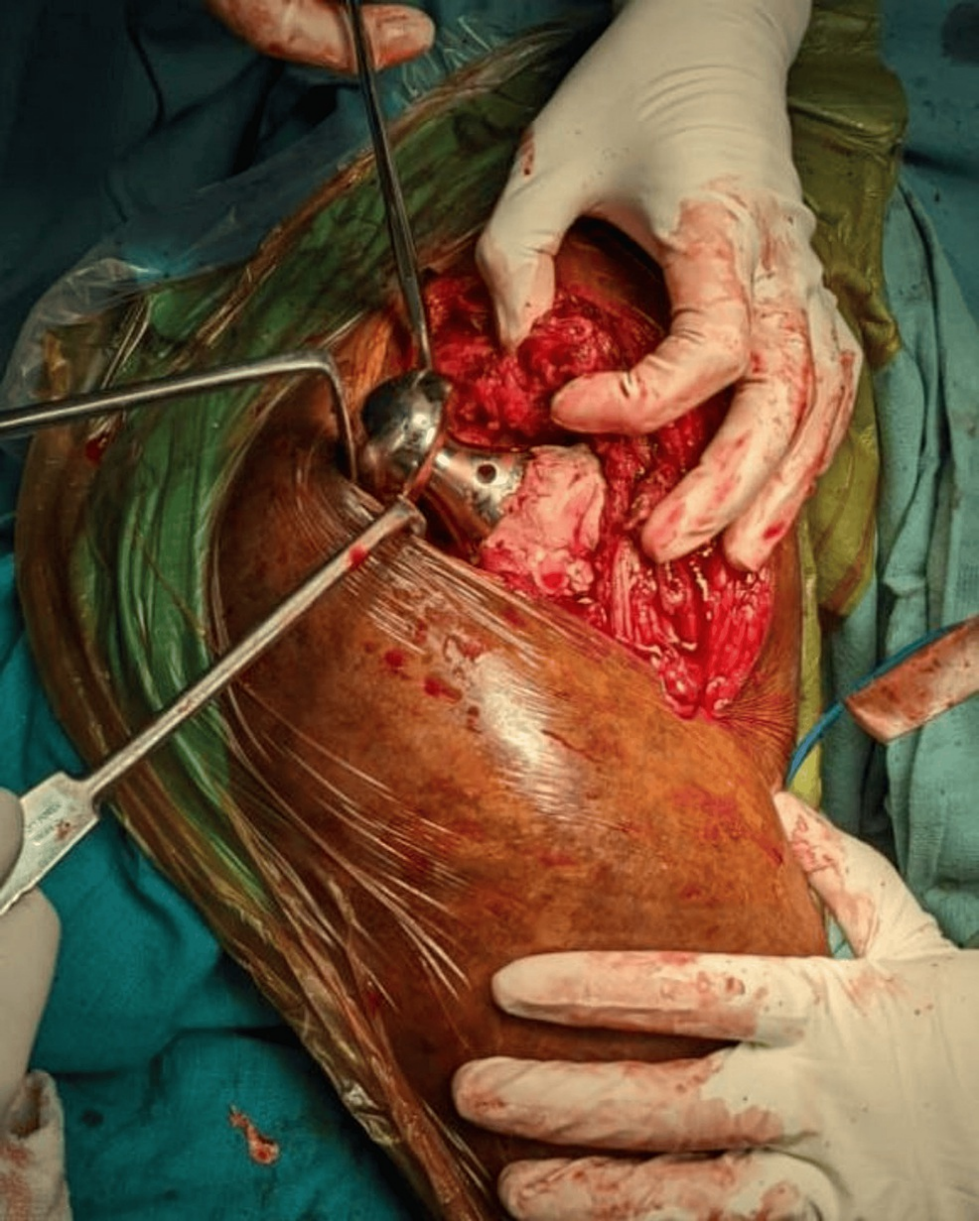
Reconstruction of the fractured calcar using cement collar

Bone cementing was performed by using a standard technique, and a bipolar prosthesis was introduced up to the predetermined length measured during the trial reduction to maintain the desired vertical offset. Reconstruction of the fractured calcar involved the application of a cement collar during prosthesis insertion (Figure 6).

**Figure 7:**
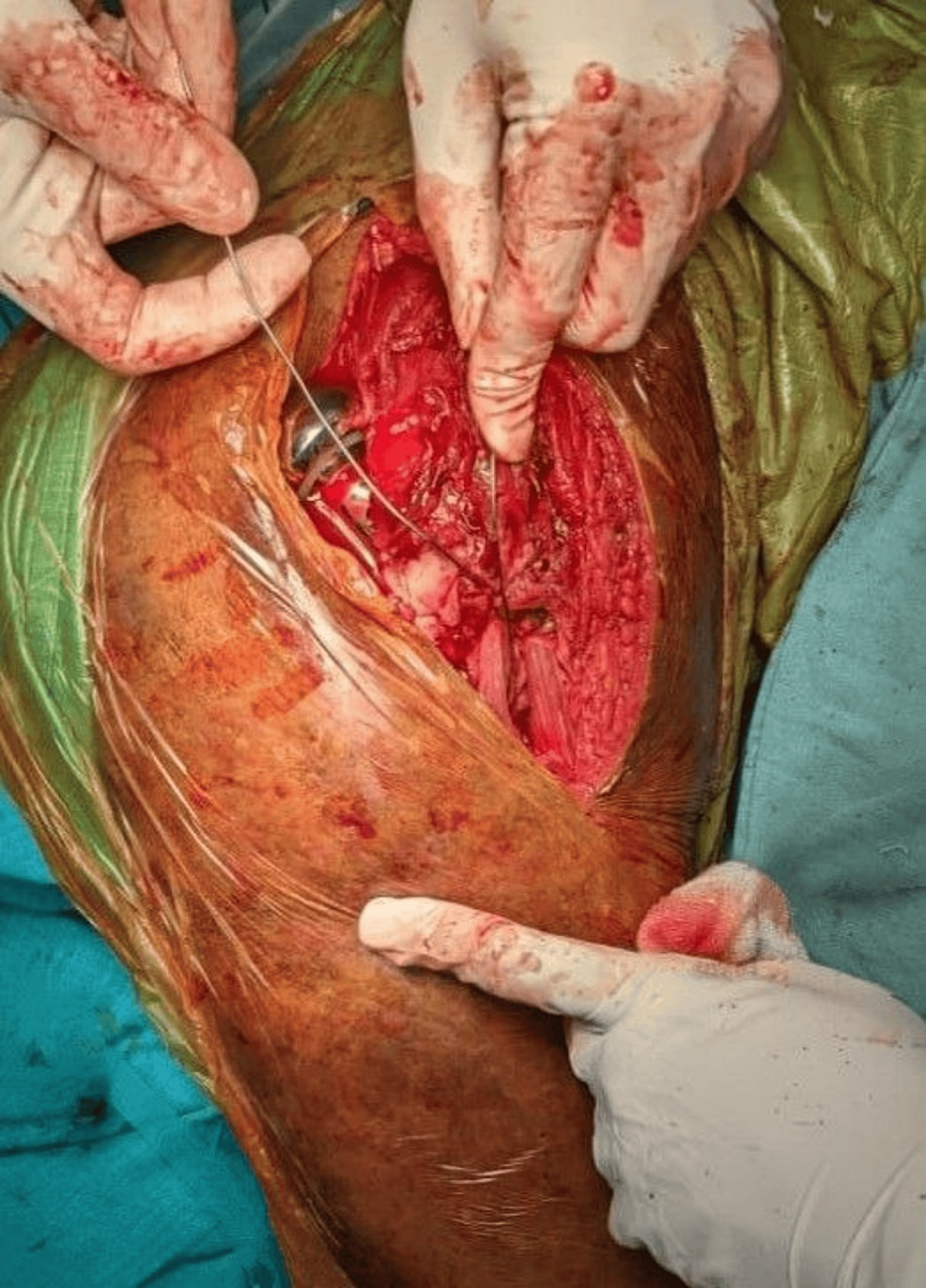
Tension-band wiring of greater trochanter

After the bone cement was set, the prosthesis was reduced to the hip joint. Greater trochanter reconstruction was achieved through tension-band wiring or purse-string sutures with 5-0 Ethibond (Figure 7).

**Figure 8:**
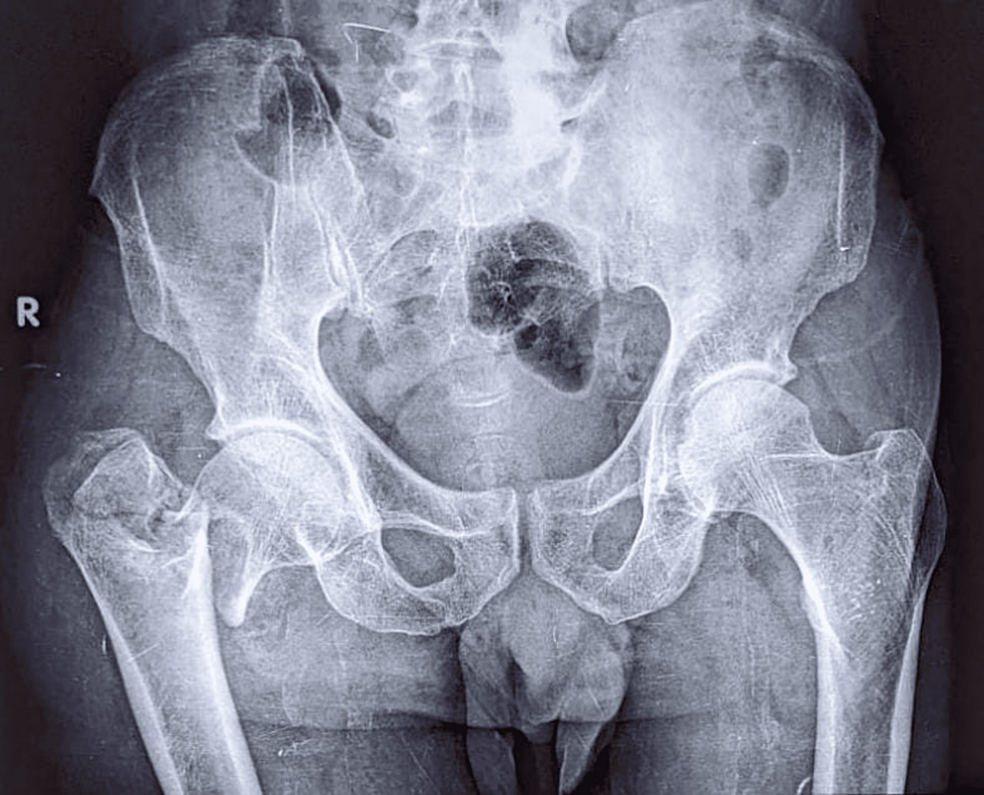
Preoperative radiograph showing unstable intertrochanteric fracture of the right femur

**Figure 9:**
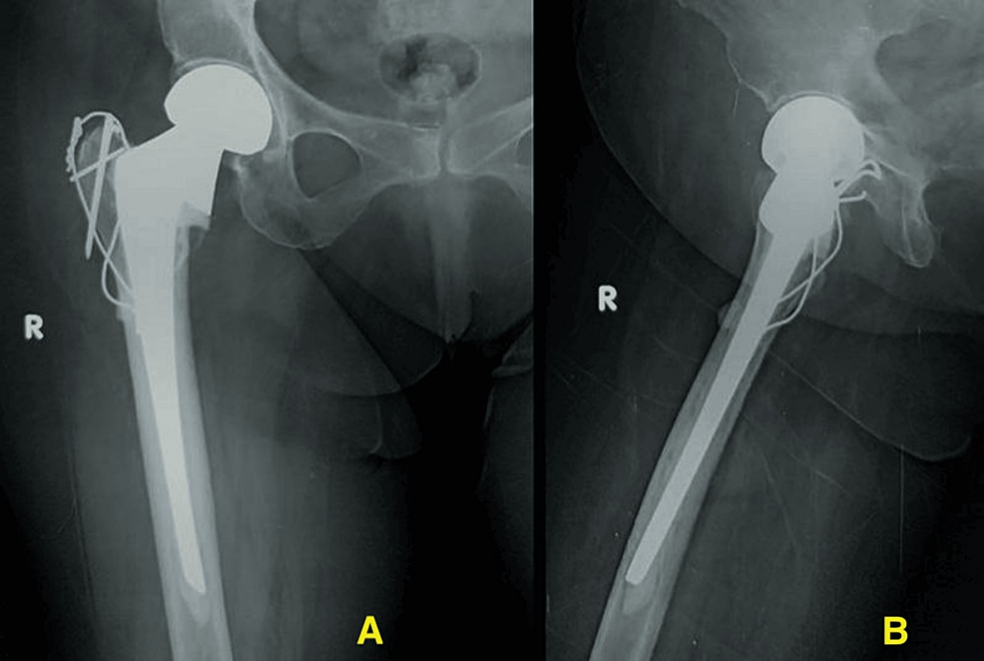
Postoperative radiographs showing cemented bipolar prosthesis and fixation of greater trochanter with tension-band wiring 9A: Anteroposterior view of the right hip with thigh showing cemented bipolar prosthesis with tension-band wiring of the greater trochanter. 9B: Lateral view of the right hip with thigh

Wound closure was performed in layers over a negative suction drain, which was removed during the first dressing change after 48 hours. Postoperative radiographs were obtained at this juncture (Figure 8, 9).

Patients were mobilized to a sitting position on the second day, and in-bed physiotherapy was initiated, with weight-bearing encouraged based on individual pain tolerance. However, activities such as sitting cross-legged and squatting were restricted. Suture removal occurred on the 14th postoperative day. Follow-up evaluations were conducted at six weeks, three months, six months, and one year. Clinical and radiological analyses were performed at each follow-up, focusing on the stem position, stem loosening, and periprosthetic fractures. The data collected was analyzed using SPSS Statistics version 21 (IBM Corp., Armonk, NY). Categorical data were presented in proportions.

## Results

The total sample comprised 20 individuals, with a mean age of 71.65 ± 8.83 years. Of these, 12 (60%) were females and eight (40%) were males. A significant proportion of participants (n=9, 45%) exhibited no comorbidities. Among those with comorbid conditions, three participants (15%) had hypertension, one participant (5%) had diabetes, and seven (35%) had multiple comorbidities. Incident categorization revealed that among the participants, 13 (65%) had experienced falls while walking, while seven participants (35%) had reported falls attributed to incidents involving a bike, cot, or chair. As for the distribution of affected sides, the left side was affected in 10 cases (50%) while it was the right side in the remaining 10 (50%).

Of note, 17 (85%) were classified as type II fractures, while the remaining three cases (15%) were classified as type III fractures (according to Boyd and Griffin's classification). The distribution of associated injuries in the study cohort revealed that 18 cases (90%) had none. Among the remaining 10%, one patient (5%) had a left distal radius fracture, and another patient (5%) had a right proximal humerus fracture. According to Singh's Index [8], the study cohort exhibited the following distribution of grades: grade 3 was observed in five cases (25%), grade 4 in 11 cases (55%), and grade 5 in four cases (20%), indicating that a majority of patients had osteoporosis.

The prosthesis used in the operative procedure was a nonmodular bipolar prosthesis with cement in all cases. Intraoperatively, the mean blood loss was 275 ± 57.35 ml (range: 200-350 ml). The average hospital stay was 6.55 ± 1.95 days (range: 4-12 days). The mean operative duration was 80.25 ± 10.19 minutes (range: 60-100 minutes). Postoperatively, 14 out of 20 patients (70%) did not require any blood transfusion, while six patients (30%) received one unit of blood each.

Postoperatively, two patients (10%) exhibited abductor weakness, while one patient (5%) experienced shortening. During the postoperative period and subsequent follow-up, one patient (5%) developed a superficial wound infection, which was effectively treated with regular dressings and antibiotic therapy. No instances of deep wound infection were noted in the postoperative or follow-up period. Additionally, there were no occurrences of prosthesis dislocation, sciatic nerve palsy, or deep vein thrombosis during the follow-up assessments. Notably, one patient (5%) deceased during the follow-up period due to reasons unrelated to the surgery.

Harris Hip Score distribution among the patients revealed the following categories: excellent (90-100) score was observed in four cases (21%), good (81-90) in 10 cases (52.7%), fair (71-80) in four cases (21%), and poor (<70) in one case (5.3 %). The Sociodemographic characteristics and Harris Hip Scores of the cohort are depicted in Table 1.

**Table 1:** Sociodemographic characteristics and clinical outcome of study participants (N=20)

Variables	Categories	Frequency (%)
Age, years	60-69	9 (45)
	70-79	7 (35)
	>80	4 (25)
Gender	Male	8 (40)
	Female	12 (60)
Comorbidities	Hypertension	3 (15)
	Diabetes mellitus	1 (5)
	Multiple comorbidities	7 (35)
	None	9 (45)
Mode of Injury	Fall while walking	13 (65)
	Fall from bike, cot, or chair	7 (35)
Side of injury	Left	10 (50)
	Right	10 (50)
Type of fracture (Boyd and Griffin)	Type II	17 (85)
	Type III	3 (15)
Associated injuries	None	18 (90)
	Distal radius fracture	1 (5)
	Proximal humerus fracture	1 (5)
Singh’s Index of Osteoporosis	Grade 3	5 (25)
	Grade 4	11 (55)
	Grade 5	4 (20)
Harris Hip Score	Excellent (90-100)	4 (21)
	Good (81-90)	10 (52.7)
	Fair (71-80)	4 (21)
	Poor (<70)	1 (5.3)

## Discussion

The unstable type constitutes about 50% of femoral intertrochanteric fractures, and conservative treatment in the elderly often results in complications and a high mortality rate [9]. Surgical intervention has been shown to reduce these risks; however, the optimal surgical approach remains a matter of controversy. The intricate nature of these fractures in elderly patients with osteoporosis poses significant challenges, increasing the likelihood of morbidity and mortality. Well-reduced, stable fractures with ideal implant placements exhibit high union rates but unstable fractures with comminution, suboptimal fixation, and poor bone quality in elderly patients may lead to failure rates as high as 56% [10]. Weak and osteoporotic bones in the elderly provide suboptimal support for screws, resulting in early biomechanical failure, leading to collapse and femoral head migration. This can cause limping due to the shortening of the abductor muscle lever arm [10].

Internal fixation, while a common approach, is associated with many complications, including implant cut-out from the femoral head, potentially resulting in profound functional disabilities. These findings emphasize the critical consideration required when choosing the surgical approach for unstable intertrochanteric fractures in the elderly, and the fracture type, bone quality, and potential complications associated with each method should be carefully considered [11]. Hemiarthroplasty is a frequently utilized alternative that provides stability, enables immediate full weight-bearing, and circumvents many complications associated with internal fixation. Cemented fixation is advantageous for attaining initial implant stability and facilitating rapid rehabilitation [10].

In 1987, Green et al. [12] employed bipolar hemiarthroplasty for unstable intertrochanteric fractures, which yielded positive outcomes. Similarly, in 2010, Sancheti et al. [6] achieved favorable functional results with bipolar hemiarthroplasty. In our study, we predominantly utilized standard nonmodular bipolar prostheses. Based on the findings of Yoo et al. [13] and Sivabalan et al. [14], cemented bipolar hemiarthroplasty with medial calcar augmentation has emerged as a viable option in elderly patients with osteoporosis. Notably, our study predominantly involved cemented bipolar hemiarthroplasty, consistent with the above studies. The mean age of our patients (71 years) was consistent with the findings of Sancheti et al. [6] and Sinno et al. [15]. A significant proportion of proximal femoral fractures arise from osteoporosis or minor trauma [3,4,5]. Our study identified domestic slip and fall as the primary modes of injury in approximately 65% of cases. Notably, our male-to-female ratio was approximately 0.6:1, and the higher incidence among females could be attributed to hormonal imbalances in postmenopausal age, inadequate hormonal replacement therapy, and the prevalence of malnutrition in our region.

The operative metrics in our study - a mean operative time of 80 minutes and mean blood loss of 275 ml - were comparable to Karthik et al.'s [16] findings. Kim et al.'s [17] assertion that internal fixation in osteoporotic patients may lead to a failure rate exceeding 50% aligns with our observation of senile osteoporosis in most patients, supporting the consideration of hemiarthroplasty as a viable option. Reconstruction of the greater trochanter has emerged as a crucial step in maintaining hip joint stability, with tension-band wiring performed in the majority of our cases. In a study by Grimsurd et al. [18], involving cemented bipolar hip arthroplasty for unstable intertrochanteric fractures, a relatively low complication rate was reported. Our patients experienced no complications such as pressure sores, pneumonia, or deep vein thrombosis, likely attributed to early partial weight-bearing protocols, progressing to full weight-bearing within a mean duration of 13.05 ± 3.37 days.

The assessment of outcomes using the Harris Hip Score revealed that 73% of our patients achieved good-to-excellent results. Comparable studies by Sancheti et al. [6] and Rodop et al. [19] reported good and excellent results of 71% and 82 %, respectively, further substantiating the promising outcomes of hemiarthroplasty in unstable intertrochanteric fractures.

This study has a few limitations, including its small sample size and relatively short duration. Additionally, the absence of a control group comprising patients treated with osteosynthesis techniques hinders the ability to engage in a comprehensive comparative analysis.

## Conclusions

Based on our findings, primary hemiarthroplasty is a reliable and effective approach for managing unstable intertrochanteric fractures in elderly individuals, delivering a stable, pain-free, and mobile joint with an acceptable complication rate. The crucial surgical steps of reconstructing the greater trochanter for a robust abductor mechanism and employing medial calcar augmentation with cement to maintain a vertical offset contribute significantly to the success of the procedure. Importantly, primary hemiarthroplasty not only ensures adequate fixation but also facilitates early mobilization in elderly patients, mitigating postoperative complications, such as pneumonia, venous thrombosis, pulmonary embolism, and decubitus ulcers.

## References

[1] Banan H, Al-Sabti A, Jimulia T, Hart AJ (2002). The treatment of unstable, extracapsular hip fractures with the AO/ASIF proximal femoral nail (PFN)--our first 60 cases. Injury.

[2] Al-yassari G, Langstaff RJ, Jones JW, Al-Lami M (2002). The AO/ASIF proximal femoral nail (PFN) for the treatment of unstable trochanteric femoral fracture. Injury.

[3] Koval KJ, Zuckerman JD (1998). Hip fractures are an increasingly important public health problem. Clin Orthop Relat Res.

[4] Lin TC, Wang PW, Lin CT, Chang YJ, Lin YJ, Liang WM, Lin JC (2021). Primary hemiarthroplasty after unstable trochanteric fracture in elderly patients: mortality, readmission and reoperation. BMC Musculoskelet Disord.

[5] Sniderman J, Vivekanantha P, Shah A, Safir O, Wolfstadt J, Kuzyk P (2023). Hemiarthroplasty for unstable intertrochanteric hip fractures: a matched cohort study. J Arthroplasty.

[6] Sancheti Kh, Sancheti P, Shyam A, Patil S, Dhariwal Q, Joshi R (2010). Primary hemiarthroplasty for unstable osteoporotic intertrochanteric fractures in the elderly: a retrospective case series. Indian J Orthop.

[7] Kumar Gn K, Meena S, Kumar N V, S M, Raj Mk V (2013). Bipolar hemiarthroplasty in unstable intertrochanteric fractures in elderly: a prospective study. J Clin Diagn Res.

[8] Singh M, Nagrath AR, Maini PS (1970). Changes in trabecular pattern of the upper end of the femur as an index of osteoporosis. J Bone Joint Surg Am.

[9] Tu DP, Liu Z, Yu YK, Xu C, Shi XL (2020). Internal fixation versus hemiarthroplasty in the treatment of unstable intertrochanteric fractures in the elderly: a systematic review and meta-analysis. Orthop Surg.

[10] Solanki PB, Panchal HC (2021). Role of hemiarthroplasty in comminuted intertrochanteric fractures in elderly. Int J Orthop Sci.

[11] Singh J, Kumar D, Kumar S (2022). Functional outcome of hemiarthroplasty of the hip for unstable intertrochanteric fractures of the femur in elderly patients: a prospective study. Cureus.

[12] Green S, Moore T, Proano F (1987). Bipolar prosthetic replacement for the management of unstable intertrochanteric hip fractures in the elderly. Clin Orthop Relat Res.

[13] Yoo JI, Cha YH, Kim KJ, Kim HY, Choy WS, Hwang SC (2018). Comparison between cementless and cemented bipolar hemiarthroplasty for treatment of unstable intertrochanteric fractures: systematic review and meta-analysis. Hip Pelvis.

[14] Sivabalan T, Thirunarayanan V, Senthil Kumar S, Ramprasath DR, Basheer S (2017). Functional analysis of cemented bipolar hemiarthroplasty with medial calcar augmentation for unstable intertrochanteric fractures in elderly. Int J Res Orthop.

[15] Sinno K, Sakr M, Girard J, Khatib H (2010). The effectiveness of primary bipolar arthroplasty in treatment of unstable intertrochanteric fractures in elderly patients. N Am J Med Sci.

[16] Karthik K, Natarajan M (2012). Unstable trochanteric fractures in elderly osteoporotic patients: role of primary hemiarthroplasty. Orthop Surg.

[17] Kim WY, Han CH, Park JI, Kim JY (2001). Failure of intertrochanteric fracture fixation with a dynamic hip screw in relation to pre-operative fracture stability and osteoporosis. Int Orthop.

[18] Grimsrud C, Monzon RJ, Richman J, Ries MD (2005). Cemented hip arthroplasty with a novel cerclage cable technique for unstable intertrochanteric hip fractures. J Arthroplasty.

[19] Rodop O, Kiral A, Kaplan H, Akmaz I (2002). Primary bipolar hemiprosthesis for unstable intertrochanteric fractures. Int Orthop.

